# A unique case of food restriction and OCD diagnosed as PANDAS and a review of the literature

**DOI:** 10.3389/fpsyt.2025.1704296

**Published:** 2026-01-27

**Authors:** Amal Y. Kentab, Rolan Bassrawi

**Affiliations:** 1 Division of Pediatric Neurology, Department of Pediatrics, College of Medicine, King Saud University, Riyadh, Saudi Arabia; 2 Department of Pediatrics, King Saud University Medical City, King Saud University, Riyadh, Saudi Arabia

**Keywords:** PANDAS, OCD, eating disorders, streptococcal infection, PANS, behavioral disorder

## Abstract

Pediatric autoimmune neuropsychiatric disorder associated with streptococcal infections (PANDAS) is recognized as a significant causative element in the emergence of childhood-onset obsessive-compulsive disorder (OCD) and movement disorders, including tic disorders, and choreiform movement. It is associated with various behavioral and psychiatric manifestations in children, such as separation anxiety disorder, body dysmorphic disorder, attention deficit hyperactivity disorder, and eating disorders. We describe a unique case of progressive weight loss in a previously healthy 13-year-old child who had a sudden and dramatic onset of refusal to eat, induction of vomiting, emotional liability, anxiety disorder, and OCD over 6 months following fever and pharyngitis. His workup showed an elevation of antistreptolysin O (ASO) titer with a negative throat swab. PANDAS was considered. His irritability improved after a course of antibiotics, and abatement of all symptoms and progressive weight gain occurred after a course of intravenous immunoglobulin (IVIG) followed by monthly benzathine penicillin injections. This report could help to increase awareness among physicians, highlight the importance of early diagnosis, and promote treatment of PANDAS among patients who present with a combination of eating disorders and OCD.

## Introduction

Pediatric autoimmune neuropsychiatric disorder associated with streptococcal infections (PANDAS) is recognized as a significant causative element in the emergence of childhood-onset obsessive-compulsive disorder (OCD) and tic disorders, including Tourette’s disorder (1, 2). Obsessive-compulsive symptoms have been documented in approximately 75% of children diagnosed with Sydenham’s chorea, as well as in other investigations pertaining to childhoodonset obsessive-compulsive disorder (OCD) and Tourette’s syndrome (3–6). As a result, a distinct subset of children has been identified, whose clinical progression is marked by sudden and significant worsening of symptoms in conjunction with group A beta-hemolytic streptococcal (GABHS) infection (6). The presence of either a positive throat culture accompanied by elevated antistreptococcal titers (showing a twofold increase within one month after infection) or high titers along with a recent occurrence of scarlet fever or pharyngitis can be noticed. Since 2012, PANDAS has been considered a subgroup of pediatric acute onset neuropsychiatric syndrome (PANS) (7), and OCD or severe food restriction is considered as a major criterion. Other criteria include the presence of various behavioral and psychiatric manifestations such as separation anxiety disorder, body dysmorphic disorder, and attention deficit hyperactivity disorder (ADHD).

Molecular mimicry is the basic mechanism of this autoimmune disorder (8). Immunosuppressive agents are effective during the acute phase (9), and long-course antibiotic therapy (2–6 weeks), prophylactic intramuscular benzathine penicillin (monthly), and adenotonsillectomy have been reported to produce long-lasting results in some patients (10, 11) Psychotropics compounds and psychotherapy therapy have been considered to treat neuropsychiatric manifestations at various times during the illness (12). We describe a unique case of progressive weight loss in a previously healthy 13-year-old child who had a sudden and dramatic onset of refusal to feed, induction of vomiting, emotional liability, anxiety disorder, and OCD following a GABHS throat infection.

## Case presentation

### Patient information

A 13-year-old Saudi boy presented to the outpatient pediatric clinic at King Khalid University Hospital, Riyadh, Saudi Arabia, for evaluation of progressive weight loss due to an abrupt onset of food restriction in the form of refusal to eat, induction of vomiting, anxiety disorder with continuous ideation of sickness, non-specific headache, dizziness, and fatigue. The symptoms had lasted for 6 months and had recently worsened for one month. The symptoms were preceded by a history of fever, pharyngitis, vomiting, and abdominal pain for 3 days, and the patient did not receive antibiotics as a viral infection was presumed to be the cause. The patient had a recent history of travel for 3 weeks during the summer season before the onset of symptoms. There was no sudden onset of motor or vocal tics, visual or auditory hallucination, separation anxiety, insomnia, or nightmares. He was diagnosed with secondary nocturnal enuresis 3 months before the problems.

According to his mother, he had lost 8.6 kg over the last 6 months mainly because of refusal to eat and nausea with induction of vomiting 3 times per week. Although he had good school performance, missing school days were noted recently, which were secondary to his illness. He was evaluated by both a psychologist and psychiatrist, who diagnosed him with generalized anxiety disorder and obsessive-compulsive disorder (OCD) in the form of fear of dirt, fear of contamination, preoccupation with body and hand washing, checking rituals, and fear of harm coming to relatives. Cognitive behavioral therapy (CBT) failed to control his symptoms, and he was started on risperidone (an antipsychotic medication) and sertraline (a selective serotonin reuptake inhibitor) but did not show much improvement. Finally, he was admitted to the general pediatric ward to treat his moderate dehydration with borderline blood pressure and to support his poor nutritional status.

He had no history suggestive of body dysmorphic disorder or ADHD. His parents noticed that he became very preoccupied with the idea of being sick and concerned about being healthy again. Around the same time that his OCD began, he suddenly developed mood lability where minor incidents would trigger variable degrees of emotional reactions. He also developed clumsiness, decreased concentration, and restlessness.

He had no previous history of emotional or behavioral problems. Furthermore, he did not possess any prior instances of sexual or physical abuse or neglect. During the early stages of life, he experienced recurring upper respiratory tract infections and pharyngitis occurring every two months throughout the winter season, without necessitating drug treatment. He had no known streptococcal throat infections, scarlet fever, rheumatic fever, or autoimmune disorder. He was a product of a full-term, uneventful pregnancy with no history of neonatal problems.

He successfully attained developmental milestones at the designated periods, and during his initial assessment, he had successfully concluded his second year of secondary education. Throughout his academic tenure, he had a commendable level of attention span, devoid of impulsivity or hyperactivity, and demonstrated exceptional coordination.

The patient had consistently resided with his parents and two female siblings who were in good health. Both parents possessed advanced degrees and were happily married. They provided attentive, affectionate, and substantial support to him. The mother exhibited good health, however, the father had diabetes and had recently been diagnosed with social phobia and depression. His paternal grandfather received a diagnosis of bipolar disorder at the age of 30. No familial history of motor or vocal tics, OCD, rheumatic fever, or Sydenham’s chorea was identified.

### Clinical findings

His appearance was characteristic of his reported age, and his pubertal stage was consistent with Tanner stage III. He exhibited amiable and collaborative behavior, consistently maintaining strong eye contact with the examiner. He had no difficulty separating from his parents to meet with the examiner. No restlessness, fidgeting, or tics were observed.

His speech was characterized by spontaneity, maintaining a consistent tempo, rhythm, and intensity, without any difficulty in articulation. He exhibited signs of anxiety, such as restlessness, agitation, panic attacks, sweating, sometimes rapid breathing and palpitation, trembling, constant worrying, and lack of patience. There was no indication of psychosis, and he refuted any thoughts of suicide or homicide. He exhibited vigilance and complete orientation, and his perception and decision-making seemed to be appropriate for his age. Neurologically, he had normal cranial nerves, motor examination, and cerebellar function with no focal deficit.

### Diagnostic assessment

A diagnosis of OCD disorder was made based on the DSM-IV criteria. Furthermore, he met the established criteria for PANDAS as a subset of PANS (6, 7). His initial laboratory studies revealed a normal hemogram, biochemistry, electrolytes, and renal and liver profiles. His erythrocyte sedimentation rate (ESR) was 21 mm/hr., and he had an elevated antistreptococcal antibody titer (antistreptolysin O) of 540 U/L (NR < 150 IU/ml). Antistreptococcal deoxyribonuclease-B test was not available. Throat culture was negative for GABHS. Electrocardiogram and ECHO were normal. Brain computerized tomography (CT) scan and magnetic resonance imaging (MRI) were unremarkable. A thyroid function test revealed low T4 and normal TSH, without clinical manifestations of hypothyroidism, which was attributed initially to his status (dehydration and electrolyte disturbance). A CT scan for paranasal sinuses was unremarkable, CSF analysis and culture were normal, and his vasculitis work-up and autoimmune panel (serum and CSF) were unremarkable (**Table 1**).

**Table 1:** Laboratory and imaging profile of the patient in this report.

Test name	Results	Reference range
White blood cell (WBC)	11.7x10^9^/L	(4.5-13.5x10^9^/L)
Neutrophil %	78.4 K/uL	(44-55 K/uL)
Lymphocyte %	16.1 K/uL	(38-42 K/uL)
Hemoglobin level (Hgb)	130 g/L	(115-145 g/L)
Mean corpuscular hemoglobin (MCH)	27.2 pg	(27.0-35.0 pg)
Mean corpuscular hemoglobin concentration (MCHC)	36.5 g/dl	(32.8-38.6 g/dl)
Platelet	220 K/uL	(140-450 K/uL)
ESR	21 mm/hr	(0-15 mm/hr)
Electrolytes	Normal	Normal
Liver profile	Normal	Normal
Total protein	78.4 g/L	(60-80 g/L)
Albumin	43.50 g/L	(38-54 g/L)
Urea	4.8 mmol/L	(2.76-8.07 mmol/L)
Creatinine	41 mcmol/L	(27-62 mcmol/L)
Hepatitis, Ebstein bare virus, cytomegalovirus, herpes virus I, and Mycoplasma serology	IgM, IgG	Non-reactive
Antistreptolysin O titer (ASO)	545 IU/ml (on admission) 434 IU/ml (6 weeks post-therapy) 406 IU/ml (8 weeks post-therapy) 450 IU/ml (9 months post- therapy)	(0-150 IU/ml)
Throat swab	Culture/Sensitivity	Negative for GABHS
T4	8.93 pmol/L 10.50 pmol/L (post-therapy)	(12-22 pmol/L)
TSH	2.44 mIU/L 2.740 mIU/L (post-therapy)	(0.25-5 mIU/L)
CSF White blood cell (WBC)	0/cumm	(0-5 cumm)
CSF Glucose	3 mmol/L	(2.22-3.89 mmol/L)
CSF Protein	0.25 g/L	(0.15-0.45 g/L)
CSF oligoclonal bands	Negative	Negative
CSF culture	Negative	Negative
CSF viral multiplex	Negative	Negative
Metabolic work-up	Unremarkable	Normal range
Autoimmune encephalitis work-up	Unremarkable	Negative
ECG, ECHO	Unremarkable	Normal
Paranasal sinus CT scan	Unremarkable	Normal
Brain MRI	Unremarkable	Normal

* GABHS; Group A ß hemolytic streptococcus. Metabolic work-up; serum ammonia, lactate, venous blood gas, tandem mass spectrometry, and urinary organic acid. Autoimmune encephalitis work-up; myelin oligodendrocyte glycoprotein, anti-N-methyl-d-aspartate receptor, and neuronal voltage-gated potassium channel antibodies.

### Therapeutic intervention

Although pharyngitis with fever started 3 days before the onset of the OCD 6 months prior, a throat culture was not done until several weeks after the onset of OCD. Upon admission, the patient did not present any symptoms of pharyngitis, and the results of his throat culture did not indicate the presence of GABHS. However, the precise etiology of the exacerbation of symptoms one month before admission remained uncertain. He received a 7-day course of Augmentin (amoxicillin + clavulanate potassium). Shortly after initiating the amoxicillin-clavulanate treatment, his agitation significantly diminished and nearly vanished, although his OCD, odd thoughts, and refusal to eat did not show any improvement.

The treatment in general is case-by-case, and also depends on the opinion of an expert. Due to the chronic course of his illness, the persistence of his symptoms (moderately to severely affected), and his current status (dehydration and loss of weight), pulse IV steroid therapy (effective during acute flare) was not an option in our patient. Oral steroid was not tried again for the same reasons, and because of his intermittent vomiting. He received intravenous immunoglobulin (IVIG) at a dosage of 1 g/kg daily for two consecutive days. The patient exhibited a favorable tolerance to the infusion, without any significant adverse effects, and was subsequently discharged on Augmentin to complete a 14-day regimen.

### Follow-up and outcomes

**Figure 1:**
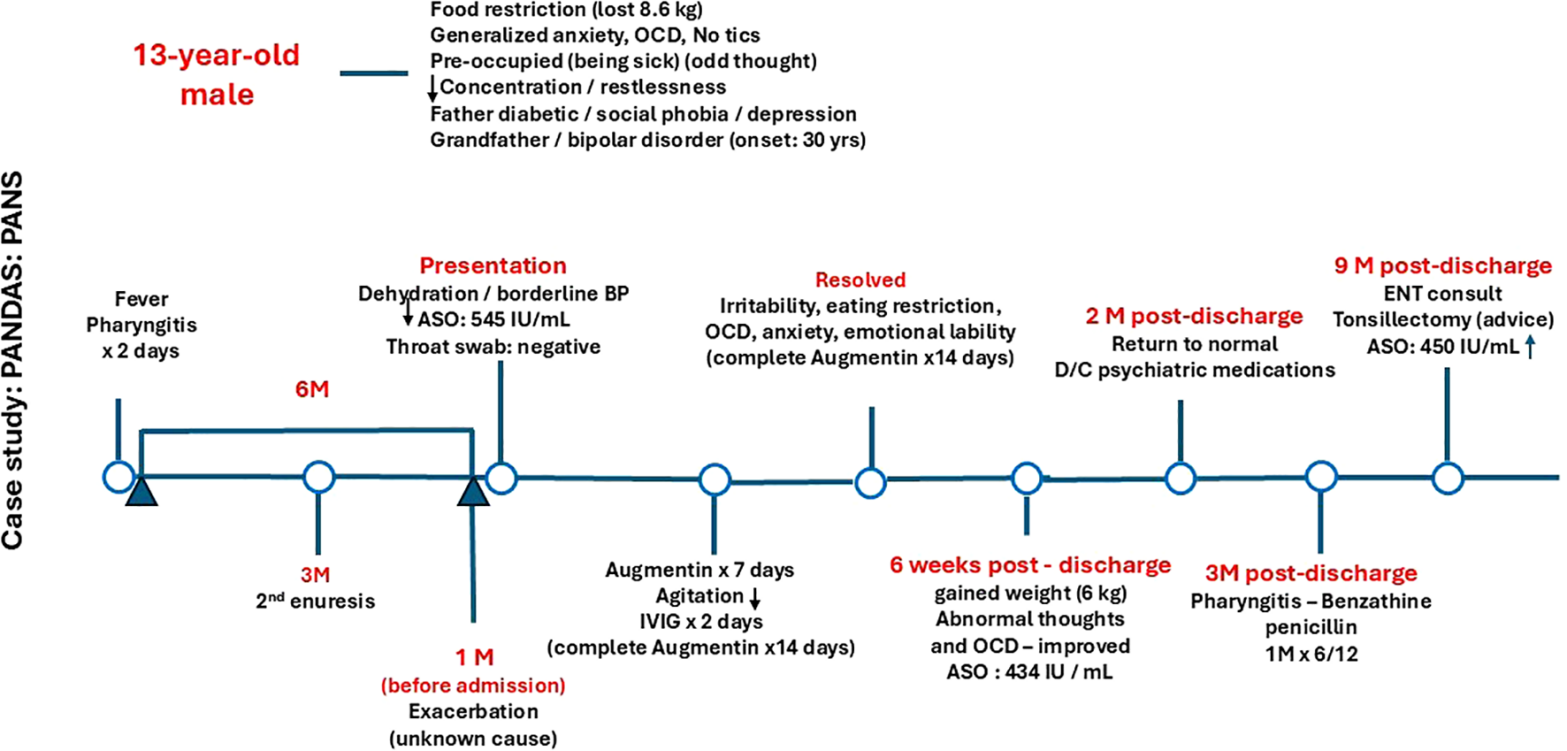
Diagnostic and patient management timeline. OCD, obsessive-compulsive disorder; ASO, Antistreptolysin O titer; BP, Blood pressure; D/C, Discontinue; IVIG, Intravenous immunoglobulin.

The patient was evaluated at the outpatient neurology clinic at 4 weeks post-discharge. His irritability, emotional lability, refusal to eat, and anxiety all resolved by 10 days post-IVIG, and he gained 6 kg within 6 weeks. Abnormal thoughts and OCD were still present but had greatly improved in comparison to the first presentation. Two months post-discharge, he returned to his normal original state, and his psychiatrist started weaning him off antipsychotic medications over 6 weeks. He was started on benzathine penicillin at 1.2 mU IM injection monthly for 6 months but he did not require further psychotropic medications or behavioral therapy. **Figure 1** highlight the patient’s timeline course.

## Discussion

Based on new diagnostic criteria (7), the inaugural PANS Consensus Conference of 2013 (13), and the diagnostic criteria of PANDAS and PANS (14), PANDAS is considered a subgroup of PANS based on the latest diagnostic criteria (7). OCD (15) or severe food restriction (16, 17) is a major criterion, in addition to the presence of other neuropsychiatric manifestations (**Table 2**). Roughly 50% of PANS patients have some degree of restrictive eating, and life-threatening consequences occur in about 17% (18).

**Table 2:** Characteristic diagnostic criteria of both PANDAS, and PANS. (Swedo SE, 1998,2012).

PANDAS Diagnostic criteria (1998)
Presence of OCD and/or tic disorder (must meet diagnostic criteria by DSM-III-R, DSM-IV, or DSM-IV-TR)
Pediatric onset (prepubertal symptoms; between age 3 and beginning of puberty)
Episodic (relapsing-remitting) course (acute or abrupt onset of symptoms or dramatic symptom exacerbations).
Temporal association between group A beta-hemolytic streptococcus infection (GABHS) and symptoms onset / exacerbation
Association with neurologic abnormalities (motoric hyperactivity, choreiform movement, and others including separation anxiety disorder, body dysmorphic disorder, and ADHD) *All five diagnostic criteria must be met

PANS Diagnostic criteria (2012)
Abrupt, dramatic onset of OCD or severely restricted food intake (<48 h). The OCD symptoms must be severe and frequent enough to meet DSM-IV criteria
Concurrent presence of additional neuropsychiatric symptoms, with *at least two of the following categories:
a. Anxiety
b. Emotional lability and/or depression
c. Irritability, aggression, and/or severely oppositional behaviors
d. Behavioral (developmental) regression
e. Deterioration in school performance
f. Sensory or motor difficulties
g. Somatic signs or symptoms, including sleep disturbances, enuresis, or urinary frequency.
Symptoms are not better explained by a known neurologic or medical disorder, such as Sydenham chorea, systemic lupus erythematosus, Tourette syndrome, or others.

Note: Comprehensive diagnostic work-up is needed to rule out mimics, and will depend on associated symptoms ( ie MRI scan, lumbar puncture, electroencephalogram, or other diagnostic tests ). Prompt treatment of GABHS infections with penicillin or other antibiotics can suppress or blunt the expected rise in streptococcal antibodies

*PANDAS*, Pediatric autoimmune neuropsychiatric disorders associated with streptococcal infections*; PANS*, Paediatric acute-onset neuropsychiatric syndrome; *OCD*, Obsessive-compulsive disorder; *ADHD*, Attention deficit hyperactivity disorder.

Restrictive eating or avoidance can include the type and amounts of both food and fluids. In the criteria for PANS, tics are not considered as a major criterion, and there are no more restrictions to the pre-pubertal age (3–12 years). For our patient, severe food restriction and OCD were major symptoms to the extent that he lost 8.6 kg over 6 months. He also had anxiety, irritability, and emotional liability with somatic symptoms of enuresis and sleep disturbances.

The proposed PANS criteria have incorporated the inclusion of eating restrictions or anorexic behaviors (19). The initial documentation of these phenomena was in a case study with a prepubescent adolescent who experienced a sudden onset of restrictive eating behavior as a result of weight gain concerns stemming from body dysmorphia. This condition was found to be temporally associated with a GABHS infection (16). Subsequently, more case studies have documented adolescents who experience abrupt beginnings of obsessional fears around contamination, worries of poisoning, choking, or vomiting, and/or sensory issues related to texture, taste, or smell (19). Body image abnormalities were observed in certain instances, but manifested later in the course of symptom progression (19). Another case report similarly emphasized the occurrence of GABHS infection prior to the development of anorexia nervosa (AN) and sudden-onset symptoms of OCD (20). Research on eating disorders in PANDAS-AN is still scarce. However, there has been some new focus on the similarities between PANS/PANDAS and avoidant/restrictive food intake disorder (ARFID) (21).

Multiple causes have been implicated for restrictive eating in PANS/PANDAS, including obsessional fears, ritualistic eating behaviors, disordered sensory hypersensitivity, difficulty swallowing, decreased appetite, or body image issues (16, 17). For many, obsessional fears are part of OCD (22), in which there are often fears of contamination/poisoning, vomiting, choking, or anaphylaxis. Disordered sensory hypersensitivity can lead to problems with tolerating particular textures, tastes, and smells. Some patients have vomiting or choking, which may indicate obsessional difficulty swallowing, and a swallowing study may be needed to rule out organic causes. Distorted body images are commonly seen in older age groups and in the presence of prolonged uncontrolled syndrome (7). Other eating disorders like ARFID, AN, or excessive eating can also be seen in PANS/PANDAS patients. Interestingly, non-specific gastrointestinal pain can be observed in PANS/PANDAS patients associated with strep or other infections (23).

In general, eating restriction can result in malnutrition, nutritional deficiency, medical complications, significant weight loss or failure to gain weight, psychosocial dysfunction, developmental delay, and gastrointestinal complications. The typical manifestations of starvation encompass symptoms such as anxiety, despair, irritability, and disruptions in sleep patterns (24). A correlation has been observed between eating disorders and autoimmune diseases, including anorexia, celiac disease, inflammatory bowel disease, lupus erythematosus, Hashimoto’s thyroiditis, systemic sclerosis, and Behcet’s syndrome. There is a link between eating disorders and a variety of illnesses on the basis of common immunological pathways, encompassing inflammatory and cytokine profiles, subsets of B and T lymphocytes, and profiles of autoantibodies. Furthermore, the interaction among the gut microbiota, immune regulation, and sex hormones offers a possible intricate mechanism that underlies eating disorders and elucidates the partially shared etiopathogenesis of eating disorders and autoimmune diseases (25). PANDAS is an autoimmune disorder where eating disorders probably can be explained by the same mechanism.

There is a possible correlation between PANDAS and Hashimoto’s thyroiditis (26, 27). Interestingly, our patient showed evidence of disturbed thyroid function test (low free T4 and normal TSH) without clinical manifestations of hypothyroidism, which, in combination with PANDAS, could possibly exacerbate the clinical picture of the eating disorder. There was no frank picture of thyroiditis, and his thyroid function test normalized later on. Commonly, a combination of psychiatric disorders and autoimmune or metabolic disorders can be observed in the family history of those with PANS/PANDAS (28).

Prior to diagnosing PANS/PANDAS based on food restriction criteria (7), it is crucial to assess all other causes of eating disorders. This is because the physical and mental effects of food restriction (24) may coincide with certain additional criteria of PANS and/or PANDAS (26), which highlights the lack of specificity of the PANS criteria in those with eating disorders. For our patient, we believe that the eating restriction was probably related to obsessional fears, abnormal thoughts, decreased appetite, swallowing issues, and vomiting as there was no evidence of disordered sensory hypersensitivity or distorted body image.

Aman et al. (26) reported the first study to assess the prevalence rates of PANS and PANDAS in pediatric patients with eating problems. Within the PANS group (n = 52), 63.5% (n = 33) exhibited both food restriction and obsessive-compulsive symptoms, whereas 13 individuals (25%) only experienced food restriction. Obsessive–compulsive symptoms were observed in only 11.5% (n = 6), and tics were observed in 19.2% (n =10) of participants. The types of eating disorders in the study were AN in 35 participants, avoidant restrictive food intake disorder (ARFD) in 10 participants, unspecified eating disorder in 4 participants, bulimia nervosa in 2 participants, and another specified eating disorder in 1 participant. The major co-occurring diagnoses were anxiety disorder (n = 29), mood disorder (n = 9), OCD (n = 5), ADHD (n = 5), and autoimmune disorder (n = 2).

In a separate investigation encompassing 136 pediatric patients with OCD, a mere 3% were identified as satisfying the PANS criteria. Among this subset, 60% reported experiencing sudden food restriction, while 100% reported abrupt symptoms of OCD (29).

For our patient, there was difficulty in diagnosing PANDAS based on the old established criteria, especially in relation to criteria 3, and 4. (**Table 2**) Abrupt onset of the disease was related to pharyngitis, although throat culture and ASO titer were not done at that time. It was not possible to identify the existence of a GABHS infection during the two neuropsychiatric exacerbations. The first one was at the onset of the disease, and the recent exacerbation or relapse was one month before admission, probably related to strep as the ASO titer was moderately elevated, but one should not forget that symptom exacerbation in PANS/PANDAS can also occur spontaneously or in response to environmental factors or other non-GABHS infections.

The patient had no adventitious movements, motoric hyperactivity, or piano-playing choreiform movements (6, 16) His symptoms were not explained by other disorders (5, 7, 30). His symptoms fit the new diagnostic criteria of PANS/PANDAS, and he responded to the initial course of antibiotics. The ASO titer during the initial phase of post-antibiotic therapy was still high, but after the IVIG/IM benzathine penicillin treatment, there were no rising antistreptococcal titers or active GABHS infection according to throat culture.

Treatment guidelines for PANS/PANDAS include: 1) removal of the source of infection with antimicrobials, 2) treatment of the neuroinflammation with immune modulators depending on severity, and 3) treatment of the symptoms with psychotherapy. Antibiotics, IVIG, or steroids can be used to treat brain inflammation and may quickly improve symptoms of bacterial infection-triggered eating disorder. Amoxicillin-clavulanate, penicillin, azithromycin, and cephalosporins are antibiotics usually used to treat streptococcal infections (10, 11). Cefdinir therapy has proven to result in improvement in recent-onset pediatric neuropsychiatric disorders (18). Immunomodulatory therapy with IVIG has been described to be an effective treatment for PANS/PANDAS patients based on multiple case reports, and case series (11). Although it has been tested in two double-blind RCTs, with the higher quality study (but testing one infusion of IVIG only) indicating low support for its efficacy (31, 32). However, in certain instances of PANS/PANDAS that do not show improvement with medication or behavioral therapy, immunomodulatory therapy with IVIG may be necessary, supported by studies using sequential infusions of IVIG that could successfully ameliorate psychological symptoms and dysfunction produced by underlying immune dysregulation in PANS/PANDAS (33, 34).

Immunomodulating therapies can be administered either individually or in conjunction with corticosteroids, rituximab, IVIG, plasmapheresis, and mycophenolate, as stated by the PANS Research Consortium in 2017 and treatment guidelines (11, 35). Steroid use may improve OCD symptoms if used early in the disease course, as it can abort or shorten flare duration. Its introduction late in the flare is less likely to result in dramatic responses and will require higher doses or more prolonged courses, thus may worsen the psychiatric symptoms. Oral steroids can be used, especially during the acute flare, but recurrent vomiting, gastritis, electrolyte disturbance, or the possibility of interaction with psychiatric medications may make this option not feasible, as happened with our patient. A combination of both medical interventions and psychotherapeutic treatments is effective in the presence of continuing eating abnormalities to minimize increased associated complications. Medical interventions include maintaining adequate nutrition (feeding tubes) and hydration, while hospitalization may be required for medically unstable patients. Psychotherapeutic treatments (36, 37) include psychotherapy such CBT, which is effective in reducing the severity of OCD symptoms, anxiety, and depression, as well as correcting the self-destructive patterns of thought and behavior in PANS/PANDAS patients. Exposure and Response Prevention (ERP) is another modality of behavior therapy that is mainly used for compulsive behaviors and anxiety disorder. It includes gradual exposure to food that causes fear and anxiety in a safe controlled environment, monitoring patient progress, and providing rewards for taking small bites of anxiety-inducing food. Family therapy is important to educate and empower families to firmly support weight gain and nutrition goals, and help build compassion and understanding of the patient’s experience. Evaluation by eating disorder specialists is also helpful mainly for therapeutic interventions and helping families maintain detailed food diaries and use weight graphs.

## Conclusion

This report increases awareness among physicians and highlights the importance of early proper diagnosis and prompt treatment of PANS/PANDAS among patients with new or sudden onset of eating restriction along with OCD and other changes in behavior, neurological, or cognitive characteristics. Implementing only psycho-therapeutic interventions is not enough for long-lasting recovery. Antibiotics or immunomodulatory treatment can be curative of eating restriction produced by PANS/PANDAS.

## Data Availability

The original contributions presented in the study are included in the article/supplementary material. Further inquiries can be directed to the corresponding author.
