# Correction: A unique case of food restriction and OCD diagnosed as PANDAS and a review of the literature

**DOI:** 10.3389/fpsyt.2026.1804289

**Published:** 2026-03-04

**Authors:** Amal Y. Kentab, Rolan Bassrawi

**Affiliations:** 1 Division of Pediatric Neurology, Department of Pediatrics, College of Medicine, King Saud University, Riyadh, Saudi Arabia; 2 Department of Pediatrics, King Saud University Medical City, King Saud University, Riyadh, Saudi Arabia

**Keywords:** PANDAS, OCD, eating disorders, streptococcal infection, PANS, behavioral disorder

Author “Rolan Bassrawi” was erroneously spelled as “Rolan Bassraway”. This is reflected in the author list, citation and copyright.

There was a mistake in **Figure 1** as published. “Genetic diagnosis of BTBGD (SLC1863)” was erroneously included below 6 weeks post-discharge. The corrected **Figure 1** appears below.

**Figure 1:**
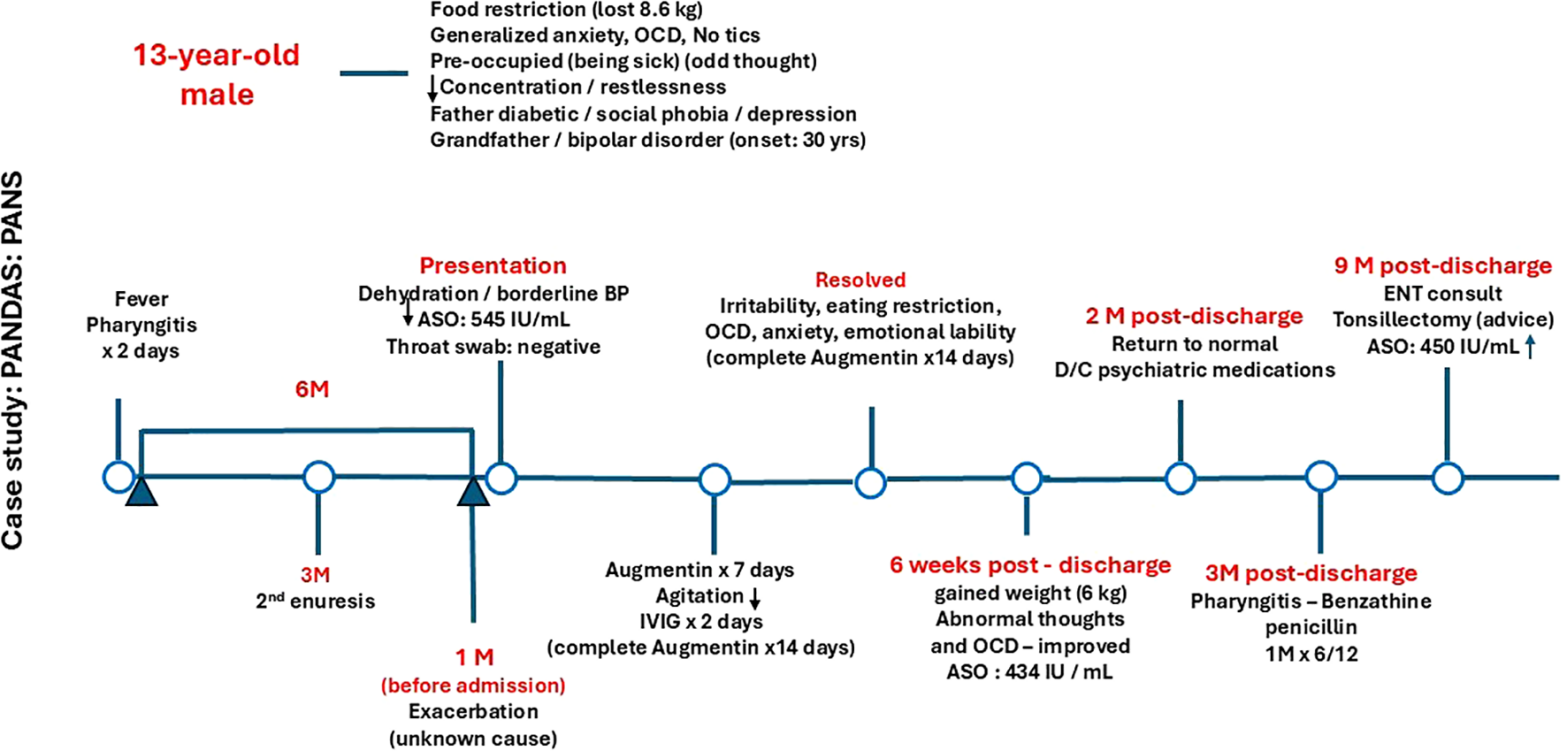
Diagnostic and patient management timeline. OCD, obsessive-compulsive disorder; ASO, Antistreptolysin O titer; BP, Blood pressure; D/C, Discontinue; IVIG, Intravenous immunoglobulin.

There was a mistake in the caption of **Figure 1**. “Antistreptolysin O titer” was erroneously written as “Antistrptolysine O titer”.

The original version of this article has been updated.

